# Age Differences in Self-Continuity in the German Socioeconomic Panel

**DOI:** 10.1093/geroni/igaa057.1257

**Published:** 2020-12-16

**Authors:** Corinna Loeckenhoff, Denis Gerstorf

**Affiliations:** 1 Cornell University, Ithaca, New York, United States; 2 Humboldt University Berlin, Berlin, Berlin, Germany

## Abstract

Self-continuity, the sense that one’s personal past, present, and future selves are meaningfully connected, is unique to human beings. Self-continuity varies across individuals with higher levels conveying benefits for mental health and well-being, physical health and health-related behaviors, as well as financial planning and moral choices (for a review see Hershfield, 2019). From a developmental point of view, self-continuity emerges over the course of childhood, but less is known about its development in adulthood. Recent evidence indicates that higher chronological age is associated with higher perceived self-continuity among healthy adults. Studies further suggest that age effects are more pronounced for more distant time intervals but fairly symmetrical for past and future (for a review see Loeckenhoff & Rutt, 2017). However, prior work has predominantly relied on U.S. convenience samples raising questions about generalizability to broader population samples as well as cross-national consistency of the findings. To address these concerns, the present study examined self-continuity in the German Socio-Economic Panel (SOEP, 2017 Innovation Sample, n = 1659, aged 18-92, M = 62.8, SD = 18.1, 53% female). In addition to replicating the previously reported positive association between age and self-continuity (r = .17, p < .001) and testing for curvilinear effects, we report on the role of temporal direction (past vs. future), temporal distance (1, 5, and 10 years), and demographic factors (i.e., gender, education, and wealth). The present findings add to the literature on adult age differences in self-continuity. Practical implications and directions for future research are discussed.

